# Seroprevalence of Antibodies against SARS-CoV-2 in Children with Juvenile Idiopathic Arthritis a Case-Control Study

**DOI:** 10.3390/jcm10081771

**Published:** 2021-04-19

**Authors:** Violetta Opoka-Winiarska, Ewelina Grywalska, Izabela Korona-Glowniak, Katarzyna Matuska, Anna Malm, Jacek Roliński

**Affiliations:** 1 Department of Paediatric Pulmonology and Rheumatology, Medical University of Lublin, Gębali 6, 20-093 Lublin, Poland; 2 Department of Clinical Immunology and Immunotherapy, Medical University of Lublin, Chodzki 4a, 20-093 Lublin, Poland; jacek.rolinski@gmail.com; 3 Department of Pharmaceutical Microbiology, Medical University of Lublin, Chodzki 1, 20-093 Lublin, Poland; iza.glowniak@umlub.pl (I.K.-G.); anna.malm@umlub.pl (A.M.); 4 Department of Diagnostic Microbiology, Independent Public Teaching Hospital No 1, Staszica 16, 20-081 Lublin, Poland; tuskama@wp.pl

**Keywords:** COVID-19, juvenile idiopathic arthritis, disease-modifying antirheumatic drugs, SARS-CoV-2

## Abstract

There is limited data on the effect of the novel coronavirus disease (COVID-19) caused by severe acute respiratory syndrome-coronavirus-2 (SARS-CoV-2) on pediatric rheumatology. We examined the prevalence of antibodies against SARS-CoV-2 in children with juvenile idiopathic arthritis (JIA) and a negative history of COVID-19 and the correlation of the presence of these antibodies with disease activity measured by juvenile arthritis disease activity score (JADAS). In total, 62 patients diagnosed with JIA, under treatment with various antirheumatic drugs, and 32 healthy children (control group) were included. Serum samples were analyzed for inflammatory markers and antibodies and their state evaluated with the juvenile arthritis disease activity score (JADAS). JIA patients do not have a higher seroprevalence of anti-SARS-CoV-2 antibodies than healthy subjects. We found anti-SARS-CoV-2 antibodies in JIA patients who did not have a history of COVID-19. The study showed no unequivocal correlation between the presence of SARS-CoV-2 antibodies and JIA activity; therefore, this relationship requires further observation. We also identified a possible link between patients’ humoral immune response and disease-modifying antirheumatic treatment, which will be confirmed in follow-up studies.

## 1. Introduction

The coronavirus disease 2019 (COVID-19) caused by severe acute respiratory syndrome-coronavirus-2 (SARS-CoV-2) presents mild disease in the majority of children [1]. However, there are a growing number of reports of severe complications after SARS-CoV-2 infection in children, specifically a Kawasaki-like disease named pediatric multisystem inflammatory syndrome temporally associated with SARS-CoV-2 (PIMS-TS) [2]. As the symptoms of SARS-CoV-2 infection in children may be non-specific and difficult to distinguish from other respiratory or non-respiratory viruses [1], children less frequently present with symptoms meeting the case definition for COVID-19 required to qualify for testing. Therefore, although collated European data show (as of 10 August 2020), children aged <10 years accounted for 1.9% of cases and 3.7% at the age of 10–19 [3], the true incidence may be higher.

There are very limited data about the infection rate and disease course in patients with rheumatic disease and almost no data regarding pediatric rheumatology patients, including juvenile idiopathic arthritis (JIA).

In the Beesley et al. study [4], based on the COVID-19 European patient registry (EPR) of 4336 adult and pediatric patients with rheumatic, autoimmune, and autoinflammatory conditions from 58 countries, COVID-19 has been diagnosed in 2.9% with only 10 adults and one child admitted to hospital. An investigation by Michelena et al. [5] of the incidence of COVID-19 in a cohort of 959 adult and pediatric patients with rheumatic diseases treated with disease-modifying antirheumatic drugs (DMARDs), based on a telephone survey and electronic health records review, identified 11 confirmed SARS-CoV-2 positive cases in the adult cohort and none in the pediatric. COVID-19 incidence rates of the rheumatic patient cohort were very similar to that of the general population.

Current Paediatric Rheumatology European Association (PRES) [6] recommendations advise that children with rheumatic diseases on medication continue all therapies, including methotrexate and biologics, as usual. It is generally known that uncontrolled disease and high disease activity place patients with JIA at higher risk of infections [7].

Nonetheless, the precise role of SARS-CoV-2 infection and its impact on disease activity and treatment in children with rheumatic diseases remains unclear.

SARS-CoV-2 serology is anticipated to be particularly valuable for prevalence surveys as serological testing can potentially detect prior infection, regardless of symptom or hospitalization history [8]. In general, the determination of antibodies is considered to be highly sensitive and specific; for example, the specificity for IgG (ELISA) is estimated at 91.9% and 73.0% for IgA [9]. Analysis of the humoral response to SARS-CoV-2 infection showed the presence of antibodies from the second day of the disease [10]. The peak concentration of IgM and IgA antibodies occurs in the 2nd week and decreases later. IgG response occurs at the same time, but the highest level is reached in the 3rd week and then decreases slowly over the next weeks [10].

The objective of this study was to examine the prevalence of anti-SARS-CoV-2 antibodies in children with JIA and a negative history of COVID-19. An exploratory aim of the study was to investigate the correlation of the presence of these antibodies with disease activity measured by the juvenile arthritis disease activity score (JADAS) [11].

## 2. Materials and Methods

### 2.1. Study Group

All subjects were diagnosed and treated in the Department of Paediatric Pulmonology and Rheumatology, Medical University of Lublin (Poland), without symptomatic history of COVID-19.

The JIA group included children with oligoarthritis (*n* = 29), polyarthritis with positive rheumatoid factor (RF) (*n* = 5), polyarthritis with negative RF (*n* = 13) and psoriatic arthritis (*n* = 3), enthesitis-related arthritis (*n* = 10), and systemic arthritis (*n* = 2). All patients diagnosed with JIA were on DMARDs, single or combination: conventional synthetic (methotrexate, sulfasalazine, hydroxychloroquine) or biologic (etanercept, adalimumab, tocilizumab). In the group treated with sulfasalazine or hydroxychloroquine, there was no concomitant administration of biological DMARDs, but in the group treated with methotrexate, there were 24 patients treated with biological DMARDs. There was no patient without treatment in the JIA group. Patients with JIA in remission without treatment were excluded from the study because, in this group, the disease activity (the indicator studied) was, by definition, low.

The control group included 32 healthy children of health workers. We excluded children taking medication affecting the immune system; reporting symptoms of infection in the last three months before the study; or patients with diagnosed chronic diseases, such as allergies, inflammatory, autoimmune, or oncological diseases. As in the JIA group, also in the control group, none of the patients had a history of COVID-19 symptoms.

Sera were obtained from a total of 104 patients (62 JIA patients and 32 controls) who were admitted to our department between 1 June to 30 September 2020. Serum samples were obtained during routine laboratory tests. Inflammatory markers, including the erythrocyte sedimentation rate (ESR) and C-reactive protein (CRP), were examined during routine outpatient visits. Clinical data were extracted from the electronic medical record.

In patients with JIA, the disease activity was estimated with the use of the juvenile arthritis disease activity score 71 (JADAS 71) [11]. The JADAS 71 includes the following four measures: physician’s global assessment of disease activity (PhGA) and parent global assessment of well-being (PGA), measured on a 0–10 visual analog scale (VAS) where 0 = no activity and 10 = maximum activity; erythrocyte sedimentation rate (ESR), normalized to a 0 to 10 scale; and a count of joints with active disease [11].

### 2.2. Detection of Anti-SARS-CoV-2 Antibodies

ELISA based tests for anti-SARS-CoV-2 IgA and IgG were from Euroimmun (Lubeck, Germany). These tests were used per manufacturer’s instructions. Results were calculated as: absorbance value of the sample divided by absorbance value of the calibrators and expressed as extinction ratio. We utilized the manufacturer’s interpretation of the ratio with samples < 0.8 classified as no antibody present, 0.8 ≤ 1.1 indeterminate, and ≥1.1 containing antibodies. These ELISA tests were for antibodies against the S1 subunit/domain of the spike protein of SARS-CoV-2.

### 2.3. Compliance with Research Ethics Standards

All patients and parents or legal guardians were informed in detail in oral and written form about the course, aims, and scope of the conducted research. All patients over 16 years and parents or guardians signed an informed written consent to participate in the study. The study was carried out in compliance with the Declaration of Helsinki. The study design was approved by the Bioethics Committee at the Medical University of Lublin (KE-0254/236/2020).

### 2.4. Statistical Analyses

Results from measurable parameters are presented as the mean, median, minimum and maximum values, and standard deviation. Nonmeasurable parameters are presented as means of count and percentage. The normal distribution of variables was checked using the Shapiro–Wilk test. The Student t test was used to compare independent variables, and the Mann–Whitney U test was used for intergroup comparisons. Differences between more than two groups were analyzed with the Kruskal–Wallis test, ANOVA, and multiple comparisons of mean ranks (as post hoc analysis) with the Bonferroni correction. The associations between pairs of variables were assessed with Spearman’s rank correlation. Statistical significance was considered at *p* < 0.05. The statistical analysis was carried out using Statistica 13.3 software (StatSoft, Kraków, Poland).

## 3. Results

### 3.1. Baseline Characteristics of the Patients

In total, 62 patients with JIA (aged 2–18 years), diagnosed according to ILAR criteria [12] (study group), and 32 healthy children (aged 1–18 years) as a control group were included. Baseline demographic and clinical characteristics of the studied groups are shown in Table 1.

A similar prevalence for SARS-CoV-2 IgA and IgG antibodies was demonstrated in JIA patients and the control group (Table 1). A total of eight JIA patients had a positive result of one of the antibodies: IgM or IgG; one patient had both positive antibodies.

### 3.2. Disease Activity in JIA Patients Seropositive and Seronegative for Anti-SARS-CoV-2

The clinical features of JIA patients who were seropositive and seronegative for SARS-CoV-2 antibodies were compared.

Significantly higher disease activity, according to JADAS 71, was demonstrated in patients who were seropositive for anti-SARS-CoV-2 IgA compared to those who were seronegative (Table 2). Nevertheless, the level of significance was rather low (*p* = 0.044), and none of the JADAS 71 components showed significant differences (PGA and active joint number; both *p* = 0.09, ESR *p* = 0.19, PhGA *p* = 0.15) (Table 2). Such a relationship was not demonstrated for anti-SARS-CoV-2 IgG. 

Neither other markers of disease activity (CRP), nor the type of therapy, differed statistically significantly between the groups (Table 2).

Correlations between anti-SARS-CoV-2 antibodies and age, disease activity measures, and JADAS 71 were also assessed. Anti-SARS-CoV-2 IgA antibodies correlated with ESR (*p* = 0.004) but not with the other parameters: age, CRP, and JADAS 71 (*p* > 0.05). There was also no correlation between these parameters and IgG antibodies.

Seropositive patients were treated with different DMARDS: two biologics, three with glucocorticoids. None of the positive patients had inactive disease (JADAS ≤ 1, [11]) (Table 3). None of these patients had a history of symptoms suggestive of COVID-19.

### 3.3. Relationship between Anti-SARS-CoV-2 Antibodies and Treatment of JIA

Next, we assessed the effect of treatment on the serological response (i.e., anti-SARS-CoV-2 antibodies) by comparing antibody ratio in patients on different treatments to those of the control group (Table 4).

Significantly lower levels of anti-SARS-CoV-2 antibodies were observed in JIA patients treated with methotrexate compared to those without methotrexate treatment and the control group (*p* = 0.014, Table 5). Otherwise, sulfasalazine-treated patients showed significantly higher antibody levels than the untreated and control groups (*p* = 0.045, Table 4). Antibody levels in JIA patients treated with biological DMARDs or systemic glucocorticoids (orally, more than 2 weeks) were similar to those who were untreated or the control group (Table 4). 

Finally, anti-SARS-CoV-2 IgA and anti-SARS-CoV-2 IgG levels were similar in JIA patients with active disease (i.e., JADAS 71 > 1) and inactive disease (JADAS 71 ≤ m1), as well as the control group (Table 5).

## 4. Discussion

Our study showed a similar prevalence for the SARS-CoV-2 IgG antibody and the SARS-CoV-2 IgA antibody in children with JIA and controls (IgG: 9.7% vs. 9.4%; IgA 4.8% vs. 6.3%, respectively). Our study was carried out from June to September 2020, where the total number of confirmed cases in Poland was 23,790 (June)–89,960 (September) and the number of cumulative cases per million inhabitants was 628 (June)–2377 (September) [13].

The durability of neutralizing antibody responses against SARS-CoV-2 after COVID-19 remains unknown [14]. Generally, antibody detection is most reliable three weeks after symptom onset or exposure, particularly in the case of IgG [8,15,16,17]. Some evidence suggests that IgM and IgG antibody levels may be higher in severe cases compared to mild or asymptomatic cases [8,18]. Unfortunately, the dynamics of the IgM and IgA antibody response in COVID-19 are not well specified. It is hypothesized that the SARS-CoV-2 IgA, IgM, and IgG isotypes are probably expressed during convalescence, and although their levels decrease over time, they can still be detected two to three months after infection [8,18]. Nevertheless, SARS-CoV-2 serology is thought to have particular value in incidence studies, as they can potentially detect prior infection regardless of symptom history [8,19].

The assessment of SARS-CoV-2 seroprevalence in children without other diseases has been reported in several studies [20,21,22]. For example, a Spanish study indicated that the prevalence of SARS-CoV-2 IgG antibodies is ~5% of the study population (randomized citizens) [21]. The seroprevalence increases throughout childhood and adolescence (i.e., 1.1% in infants younger than one year and 3.1% in those aged 5–9 years) and plateaus around 6% in people aged 45 years or older [21]. Similarly, the overall prevalence of SARS-CoV-2 IgG reported in Switzerland from April to May 2020 was 4.8%, compared to only 0.8% in children aged 5–9 years and 9.6% in teenagers aged 10–19 years [22]. Likewise, in Italy, the general seroprevalence of SARS-CoV-2 IgG was 22.6% from May to June 2020 but only 8.19% in those aged 0–19 years [20]. In comparison, we found a similar seroprevalence of SARS-CoV-2 IgA and a lower seroprevalence of SARS-CoV-2 IgG; however, we found seroprevalence was not related to the age of the patients.

We also compared the clinical features of JIA patients who were seropositive and seronegative for SARS-CoV-2 antibodies. We found patients who were seropositive for the SARS-CoV-2 IgA antibody had higher disease activity according to JADAS 71 than those who were seronegative, although both groups had few positive patients, which could have influenced the results. Additionally, none of the JADAS 71 components (ESR, PGA, PhGA, active joint number) showed statistically significant difference.

Despite this, there was no significant difference in SARS-CoV-2 IgA and SARS-CoV-2 IgG antibody levels among JIA patients with active disease (JADAS 71 >1), those with inactive disease (JADAS 71 ≤1), and healthy controls.

Seropositive for SARS-CoV-2 antibody patients with JIA were treated with different DMARDs: two with biologics, three with glucocorticoids. We would like to emphasize that, regardless of treatment, none of these patients had a history of symptoms suggestive of COVID-19.

In the Michelena et al. study [5], adult patients with rheumatoid arthritis and active disease were more likely to be classified as a suspected COVID-19 case (10.8% active vs. 3.5% non-active). Marino et al. [23] described four patients with JIA on TNF inhibitors exposed to COVID-19 family members. TNF inhibitors were discontinued in all patients for a median time of 8 weeks. None of the reported patients experienced severe COVID-19 manifestations, but despite them all being in remission at the time of contact, two developed an exacerbation later.

Nevertheless, our conclusions should be taken as preliminary. Some limitations of this study might be related to the fact that children with exacerbation of the disease more often come to follow-up visits. Additionally, the study was conducted in the summer of 2020 while the incidence of infectious cases in Poland was gradually increasing and the number of infected patients was still small.

JIA patients taking MTX have the lowest risk of serious infections, whereas treatment with the biologic agents ETN and ADA increases the risk slightly. A higher disease activity expressed by cJADAS10 is independently associated with, To the best of our knowledge, this is the first study to show a correlation between JIA disease activity and SARS-CoV-2 seroprevalence. Previous studies have monitored the course of rheumatic diseases in children throughout the pandemic. For example, a telephone survey was conducted in May 2020 among 439 children with various rheumatic diseases under immunosuppressive treatment, including 243 with JIA, 109 with autoinflammatory diseases, 51 with connective tissue diseases, and 11 with vasculitis [24]. Of them, only one patient with JIA was diagnosed with COVID-19. None, including the confirmed case, had any severe symptoms, despite more than half having household exposure [24]. Nevertheless, further research is needed to unequivocally evaluate the impact of immunosuppressive treatment in the context of COVID-19.

Indeed, we found that the type of anti-inflammatory therapy had no impact on seroprevalence in JIA patients. However, we did observe lower levels of SARS-CoV-2 IgA antibodies in patients treated with methotrexate compared to those without methotrexate treatment and the control group. Meanwhile, sulfasalazine-treated patients showed higher SARS-CoV-2 IgA antibody levels than the untreated and control groups.

So how could SARS-CoV-2 antibodies be influencing disease activity in JIA? In SARS-CoV/macaque models, the presence of the anti-spike IgG (S-IgG) antibody was shown to cause severe lung injury via an abnormal inflammation-resolving response (e.g., it promoted cytokine production and proinflammatory monocyte/macrophage recruitment) [25].

An analysis of the immune system response with multisystem inflammatory syndrome in children (MIS-C), with the complication occurring 2–4 weeks after infection or contact with SARS-CoV-2, indicates that neutralizing antibodies against SARS-CoV-2, are associated with interleukin18 (IL18) and IL16 activation; myeloid chemotaxis; and activation of lymphocytes, monocytes, and natural killer cells [22]. The possible mechanisms for immune response to SARS-CoV-2 include: antibody recognition of self-antigens (mimicry) resulting in autoantibodies; antibody recognition of viral antigens expressed on infected cells; and formation of immune complexes which activate inflammation [22].

Another explanation may be based on the observation that pediatric patients have higher serum concentrations of IL-17A and IFN-γ shortly after clinical presentation of COVID-19 [26].

In the case of JIA, treatment-naive polyarticular JIA patients were found to have enhanced responsiveness to IFN-γ stimulation compared with controls (i.e., increased STAT1 and/or STAT3 phosphorylation in subsets of CD4 T cells and classical monocytes) [27]. In addition, serum IL-17A levels are elevated in children with active JIA and decrease during the inactive phase of the disease [28]. Indeed, JIA patients with detectable levels of IL-17A have significantly higher values of JADAS 27 than those with non-detectable IL-17A [28]. These similarities may explain the association of the immune response to the SARS-CoV-2 with JIA activity. However, they could also reflect the readiness of the immune system in children with JIA to defend against the virus.

So far, there are little data on the effect of virus infection on JIA activity and response to treatment [29,30]. Our recent publication [30] indicates that history of EBV infection can have a negative effect on achieving JIA remission and may be associated with a worse response to treatment. Also, a recent study demonstrates an association between EBV and activity of juvenile systemic lupus erythematosus [31]. These results suggest that viral infections can contribute to disease activity, even if they do not directly cause development. As for EBV [30], as well as for SARS-CoV-2, we hypothesized that the virus might operate using epigenetic mechanisms.

The effect of coronaviruses on host immunity through epigenetic mechanisms is a subject of pre-pandemic and current scientific discussions [32,33]. Evidence supports a hypothesis that RNA viruses have developed intricate processes that regulate the epigenome and control host’s innate immunity [32,33].

## 5. Conclusions

Children with JIA do not show a higher seroprevalence of anti-SARS-CoV-2 antibodies compared to healthy controls. Patients with JIA seropositive for SARS-CoV-2 antibody, regardless of treatment, had no history of COVID-19 symptoms. The study showed no unequivocal correlation between the presence of SARS-CoV-2 antibodies and disease activity among children with JIA; therefore, this relationship requires further observation. The effect of DMARD treatment on the humoral response is also possible. A follow-up study to confirm these relationships is planned.

## Figures and Tables

**Table 1:** Baseline demographic and clinical characteristics of the patients.

Parameter	JIA (*n* = 62)	Control (*n* = 32)	*p*
	Median (Range)	Median (Range)	
Age (years)	12.0 (2–18)	11.0 (1–18)	0.48
ESR (mm/h)	7.5 (2–120)	4.0 (2.0–20.0)	0.19
CRP (mg/dL)	0.0 (0.0–17.9)	0.0 (0.0–0.16)	0.30
IgA anti-SARS-CoV-2 (ratio)	0.37 (0.06–2.9)	0.37 (0.1–3.58)	0.69
Positive anti-SARS-CoV-2 IgA	6 (9.7%)	3 (9.4%)	1.0
IgG anti-SARS (ratio)	0.30 (0.17–1.9)	0.29 (0.19–1.21)	0.58
Positive anti-SARS-CoV-2 IgG	3 (4.8%)	2 (6.3%)	1.0
JADAS 71	4.9 (0.0–36.0)	ND	ND
Active joint number	1.0 (0.0–25.0)		
PGA	2.0 (0.0–7.0)		
PhGA	2.0 (0.0–18.6)		
Treatment			
Biological DMARDs	30 (48.4%)	ND	ND
Adalimumab	13 (21.0%)		
Etanercept	11 (17.7%)		
Tocilizumab	6 (9.7%)		
Conventional synthetic DMARDs	62 (100%)		
Methotrexate	48 (77.4%)		
Hydroxychloroquine	5 (8.1%)		
Sulfasalazine	14 (22.6%)		
Glucocorticoids *	16 (25.8%)		

JIA, juvenile idiopathic arthritis; CRP, C-reactive protein; ESR, erythrocyte sedimentation rate; JADAS 71, juvenile arthritis disease activity score 71; PhGA, physician global assessment of disease activity; PGA, parent/patient assessment of overall well-being; ND, not determined. DMARDs, disease-modifying antirheumatic drugs; * systemic glucocorticoids (orally, more than to 2 weeks, regardless of the dose).

**Table 2:** Characteristics of JIA patients who are seropositive and seronegative for anti-SARS-CoV-2.

Parameter	IgA Anti-SARS-CoV-2				IgG Anti-SARS-CoV-2			
	Positive (*n* = 6)	Negative (*n* = 56)	Z/RR (95%CI)	*p*	Positive (*n* = 3)	Negative (*n* = 59)	Z/RR (95%CI)	*p*
JIA activity								
Age (years)	11.0 (2–15)	12.0 (2–18)	0.5	0.61	4.0 (2–13)	12.0 (2–18)	1.4	0.17
ESR (mm/h)	16.0 (2.0–120.0)	6.5 (2.0–86.0)	–1.3	0.19	7.0 (2.0–9.0)	8.0 (2.0–120.0)	0.7	0.48
CRP (mg/dL)	0.03 (0.0–1.97)	0.0 (0.0–17.9)	–0.5	0.60	0.0 (0.0)	0.0 (0.0–17.9)	1.4	0.17
JADAS 71	12.5 (3.0–36)	4.0 (0.0–32.9)	–2.01	0.044	3.3 (2.0–5.0)	5.0 (0.0–36.0)	0.5	0.64
Active joint number	2.0 (0–25)	1.0 (0–15)	–1.7	0.086	0.0 (0–2)	1.0 (0.0–25.0)	0.6	0.55
PGA	3.5 (2.0–6.0)	2.0 (0.0–7.0)	–1.7	0.089	1.0 (1.0–2.0)	2.0 (0.0–7.0)	0.7	0.46
PhGA	2.5 (2.0–6.0)	2.0 (0.0–18.6)	–1.4	0.15	2.0 (0.0–2.0)	2.0 (0.0–18.6)	0.6	0.55
Therapy								
Biological DMARDs	2 (33.3%)	28 (50%)	0.67 (0.2–2.1)	0.67	0 (0)	30 (50.8%)	(∞)	0.24
Methotrexate	2 (33.3%)	47 (83.9%)	1.0 (0.2–6.8)	1.0	2 (66.7%)	46 (78.0%)	0.9 (0.4–1.9)	0.54
Sulfasalazine	2 (33.3%)	12 (21.4%)	1.6 (0.5–5.4)	0.61	1 (33.3)	13 (22.0%)	1.5 (0.3–8.0)	0.54
Glucocorticoids	1 (16.7%)	15 (26.8%)	0.6 (0.1–3.9)	1.0	1 (33.3)	15 (25.4%)	1.3 (0.2–6.9)	1.0

JIA, juvenile idiopathic arthritis; CRP, C-reactive protein; ESR, erythrocyte sedimentation rate; JADAS 71, juvenile arthritis disease activity score 71; PhGA, physician global assessment of disease activity; PGA, parent/patient assessment of overall well-being; DMARDs Disease-modifying antirheumatic drugs.

**Table 3:** Characteristics of SARS-CoV-2 antibody positive JIA patients.

JIA Patient	Type of JIA	Treatment	Disease Activity JADAS 71	Positive Anti-SARS-CoV-2	
				IgA	IgG
1	ERA	sulfasalazine	3	+	+
2	Poli RF-	etanercept	6	+	−
3	ERA	methotrexate	9	+	−
4	Poli RF-	methotrexate glucocorticoids	5	−	+
5	Poli RF-	methotrexate	2	−	+
6	Poli RF-	methotrexate	6	+	−
7	Poli RF-	tocilizumab	36	+	−
8	ERA	sulfasalazine glucocorticoids	15	+	−

Poli RF, polyarthritis with negative rheumatoid factor (and psoriatic arthritis, ERA, enthesitis-related arthritis, Syst, systemic arthritis).

**Table 4:** Anti-SARS-CoV-2 (IgA, IgG) levels in JIA patients according to therapy type.

Parameter	IgA (Ratio)	*p*	IgG (Ratio)	*p*
	Median (Range)		Median (Range)	
Patients with biological therapy (*n* = 30)	0.37 (0.06–1.23)	0.91	0.32 (0.17–0.66)	0.85
Without (*n* = 32)	0.37 (0.08–2.93)		0.29 (0.18–1.9)	
Control (*n* = 32)	0.37 (0.1–3.58)		0.29 (0.19–1.21)	
Patients with methotrexate (*n* = 48)	0.33 (0.06–1.23)	0.014	0.30 (0.17–1.9)	0.59
Without (*n* = 14)	0.50 (0.23–2.93)		0.31 (0.21–1.32)	
Control (*n* = 32)	0.37 (0.1–3.58)		0.29 (0.19–1.21)	
Patients with hydroxychloroquine (*n* = 5)	0.21 (0.11–0.54)	0.23	0.36 (0.19–0.95)	0.84
Without (*n* = 57)	0.37 (0.06–2.93)		0.29 (0.17–1.9)	
Control (*n* = 32)	0.37 (0.1–3.58)		0.29 (0.19–1.21)	
Patients with sulfasalazine (*n* = 14)	0.49 (0.28–0.11)	0.045	0.36 (0.24–1.32)	0.31
Without (*n* = 48)	0.35 (0.06–2.93)		0.29 (0.17–1.9)	
Control (*n* = 32)	0.37 (0.1–3.58)		0.29 (0.19–1.21)	
Patients with systemic glucocorticoids (*n* = 16)	0.36 (0.18–0.98)	0.9	0.32 (0.22–1.9)	0.73
Without (*n* = 46)	0.37 (0.06–2.93)		0.29 (0.17–1.32)	
Control (*n* = 32)	0.37 (0.1–3.58)		0.29 (0.19–1.21)	

**Table 5:** Anti-SARS-CoV-2 levels in JIA patients depending on disease activity (JADAS 71).

Parameter	IgA (Ratio)	*p*	IgG (Ratio)	*p*
	Median (Range)		Median (Range)	
JADAS 71 > 1 (*n* = 40)	0.37 (0.06–2.93)	0.54	0.32 (0.18–1.9)	0.42
JADAS 71 ≤ 1 (*n* = 22)	0.36 (0.13–0.73)		0.25 (0.17–0.95)	
Control (*n* = 32)	0.37 (0.1–3.58)		0.29 (0.19–1.21)	

## Data Availability

The datasets used and/or analyzed during the current study are available from the corresponding author on reasonable request.

## Abbreviations

ARDS	Acute respiratory distress syndrome
CRP	C-reactive protein
DMARDs	Disease-modifying antirheumatic drugs
ESR	Erythrocyte sedimentation rate
ILAR criteria	International League of Associations for Rheumatology criteria for JIA
JADAS 71	Juvenile arthritis disease activity score of all 71 joints
JIA	Juvenile idiopathic arthritis
MIS	Multisystem inflammatory syndrome
PGA	Parent global assessment of well-being
PhGA	Physician’s global assessment of disease activity
VAS	Visual analog scale

## References

[1] Munro A.P.S., Faust S.N. (2020). COVID-19 in children: Current evidence and key questions. Curr. Opin. Infect. Dis..

[2] Sinaei R., Pezeshki S., Parvaresh S., Sinaei R. (2020). Why COVID-19 is less frequent and severe in children: A narrative review. World J. Pediatr..

[3] European Centre for Disease Prevention and Control (2020). Coronavirus Disease 2019 (COVID-19) in the EU/EEA and the UK—Eleventh Update: Resurgence of Cases.

[4] Beesley R.P., Costello W., Angevare S.P., Wouters C., Wulffraat N., Uzie Y. (2021). Survey of adult and paediatric rheumatology patients suggests information about COVID-19 vaccination will aid uptake. Rheumatology.

[5] Michelena X., Borrell H., Lopez-Corbeto M., Lopez-Lasanta M., Moreno E., Pascual-Pastor M., Erra A., Serrat M., Espartal E., Anton S. (2020). Incidence of COVID-19 in a cohort of adult and paediatric patients with rheumatic diseases treated with targeted biologic and synthetic disease-modifying anti-rheumatic drugs. Semin. Arthritis Rheum..

[6] PRES Update Regarding COVID-19 Vaccines in Pediatric Rheumatic Patients Published on 30 December 2020. https://www.pres.eu/clinical-affairs/guidelines.html.

[7] Batu E.D., Ozen S. (2020). Implications of COVID-19 in pediatric rheumatology. Rheumatol. Int..

[8] Bailey D., Konforte D., Barakauskas V.E., Yip P.M., Kulasingam V., Abou El Hassan M., Beach L.A., Blasutig I.M., Catomeris P., Dooley K.C. (2020). Canadian society of clinical chemists (CSCC) interim consensus guidance for testing and reporting of SARS-CoV-2 serology. Clin. Biochem..

[9] Jaaskelainen A.J., Kekalainen E., Kallio-Kokko H., Mannonen L., Kortela E., Vapalahti O., Kurkela S., Lappalainen M. (2020). Evaluation of commercial and automated SARS-CoV-2 IgG and IgA ELISAs using coronavirus disease (COVID-19) patient samples. Eurosurveillance.

[10] Cheng M.P., Yansouni C.P., Basta N.E., Desjardins M., Kanjilal S., Paquette K., Caya C., Semret M., Quach C., Libman M. (2020). Serodiagnostics for Severe Acute Respiratory Syndrome-Related Coronavirus 2: A Narrative Review. Annu. Intern. Med..

[11] Consolaro A., Giancane G., Schiappapietra B., Davi S., Calandra S., Lanni S., Ravelli A. (2016). Clinical outcome measures in juvenile idiopathic arthritis. Pediatr. Rheumatol. Online J..

[12] Petty R.E., Southwood T.R., Manners P., Baum J., Glass D.N., Goldenberg J., He X., Maldonado-Cocco J., Orozco-Alcala J., Prieur A.M. (2004). International League of Associations for Rheumatology classification of juvenile idiopathic arthritis: Second revision, Edmonton, 2001. J. Rheumatol..

[13] Roser M., Ritchie H., Ortiz-Ospina E., Hasell J. Coronavirus Pandemic (COVID-19). OurWorldInData.org. https://ourworldindata.org/coronavirus.

[14] Braun J., Loyal L., Frentsch M., Wendisch D., Georg P., Kurth F., Hippenstiel S., Dingeldey M., Kruse B., Fauchere F. (2020). SARS-CoV-2-reactive T cells in healthy donors and patients with COVID-19. Nature.

[15] Sun B., Feng Y., Mo X., Zheng P., Wang Q., Li P., Peng P., Liu X., Chen Z., Huang H. (2020). Kinetics of SARS-CoV-2 specific IgM and IgG responses in COVID-19 patients. Emerg. Microbes Infect..

[16] Yongchen Z., Shen H., Wang X., Shi X., Li Y., Yan J., Chen Y., Gu B. (2020). Different longitudinal patterns of nucleic acid and serology testing results based on disease severity of COVID-19 patients. Emerg. Microbes Infect..

[17] Zhang G., Nie S., Zhang Z., Zhang Z. (2020). Longitudinal Change of Severe Acute Respiratory Syndrome Coronavirus 2 Antibodies in Patients with Coronavirus Disease 2019. J. Infect. Dis..

[18] Lynch K.L., Whitman J.D., Lacanienta N.P., Beckerdite E.W., Kastner S.A., Shy B.R., Goldgof G.M., Levine A.G., Bapat S.P., Stramer S.L. (2020). Magnitude and kinetics of anti-SARS-CoV-2 antibody responses and their relationship to disease severity. Clin. Infect. Dis..

[19] Espejo A.P., Akgun Y., Al Mana A.F., Tjendra Y., Millan N.C., Gomez-Fernandez C., Cray C. (2020). Review of Current Advances in Serologic Testing for COVID-19. Am. J. Clin. Pathol..

[20] Pagani G., Conti F., Giacomelli A., Bernacchia D., Rondanin R., Prina A., Scolari V., Gandolfi C.E., Castaldi S., Marano G. (2020). Seroprevalence of SARS-CoV-2 significantly varies with age: Preliminary results from a mass population screening. J. Infect..

[21] Pollan M., Perez-Gomez B., Pastor-Barriuso R., Oteo J., Hernan M.A., Perez-Olmeda M., Sanmartin J.L., Fernandez-Garcia A., Cruz I., Fernandez de Larrea N. (2020). Prevalence of SARS-CoV-2 in Spain (ENE-COVID): A nationwide, population-based seroepidemiological study. Lancet.

[22] Stringhini S., Wisniak A., Piumatti G., Azman A.S., Lauer S.A., Baysson H., De Ridder D., Petrovic D., Schrempft S., Marcus K. (2020). Seroprevalence of anti-SARS-CoV-2 IgG antibodies in Geneva, Switzerland (SEROCoV-POP): A population-based study. Lancet.

[23] Marino A., Romano M., Gattinara M., Cimaz R. (2020). Patients with juvenile idiopathic arthritis on TNF inhibitors exposed to COVID-19 family members. Semin. Arthritis Rheum..

[24] Koker O., Demirkan F.G., Kayaalp G., Cakmak F., Tanatar A., Karadag S.G., Sonmez H.E., Omeroglu R., Aktay Ayaz N. (2020). Does immunosuppressive treatment entail an additional risk for children with rheumatic diseases? A survey-based study in the era of COVID-19. Rheumatol. Int..

[25] Liu L., Wei Q., Lin Q., Fang J., Wang H., Kwok H., Tang H., Nishiura K., Peng J., Tan Z. (2019). Anti-spike IgG causes severe acute lung injury by skewing macrophage responses during acute SARS-CoV infection. JCI Insight.

[26] Pierce C.A., Preston-Hurlburt P., Dai Y., Aschner C.B., Cheshenko N., Galen B., Garforth S.J., Herrera N.G., Jangra R.K., Morano N.C. (2020). Immune responses to SARS-CoV-2 infection in hospitalized pediatric and adult patients. Sci. Transl. Med..

[27] Throm A.A., Moncrieffe H., Orandi A.B., Pingel J.T., Geurs T.L., Miller H.L., Daugherty A.L., Malkova O.N., Lovell D.J., Thompson S.D. (2018). Identification of enhanced IFN-gamma signaling in polyarticular juvenile idiopathic arthritis with mass cytometry. JCI Insight.

[28] Vijatov-Djuric G., Doronjski A., Mitic I., Brkic S., Barisic N. (2017). Interleukin-17A Levels Increase in Serum of Children with Juvenile Idiopathic Arthritis. Arch. Rheumatol..

[29] Aghighi Y., Gilani Sh M., Razavi M., Zamani A., Daneshjoo K. (2007). Juvenile rheumatoid arthritis in children with Ebstein Barr virus infection. Pak. J. Biol. Sci..

[30] Opoka-Winiarska V., Grywalska E., Sobiesiak A., Rolinski J. (2020). The Impact of Epstein-Barr Virus Infection on Juvenile Idiopathic Arthritis Activity and Patient’s Response to Treatment. J. Clin. Med..

[31] Aygun D., Kuskucu M.A., Sahin S., Adrovic A., Barut K., Yildiz M., Sharifova S., Midilli K., Cokugras H., Camcioglu Y. (2020). Epstein-Barr virus, cytomegalovirus and BK polyomavirus burden in juvenile systemic lupus erythematosus: Correlation with clinical and laboratory indices of disease activity. Lupus.

[32] Schafer A., Baric R.S. (2017). Epigenetic Landscape during Coronavirus Infection. Pathogens.

[33] Chlamydas S., Papavassiliou A.G., Piperi C. (2021). Epigenetic mechanisms regulating COVID-19 infection. Epigenetics.

